# Mechanobiological Modulation of Cytoskeleton and Calcium Influx in Osteoblastic Cells by Short-Term Focused Acoustic Radiation Force

**DOI:** 10.1371/journal.pone.0038343

**Published:** 2012-06-06

**Authors:** Shu Zhang, Jiqi Cheng, Yi-Xian Qin

**Affiliations:** 1 Department of Biomedical Engineering, Stony Brook University, Stony Brook, New York, United States of America; 2 The Key Laboratory of Aerospace Medicine, Chinese Ministry of Education, Xi’an, ShaanXi, People's Republic of China; University of Texas Southwestern Medical Center, United States of America

## Abstract

Mechanotransduction has demonstrated potential for regulating tissue adaptation *in vivo* and cellular activities *in vitro*. It is well documented that ultrasound can produce a wide variety of biological effects in biological systems. For example, pulsed ultrasound can be used to noninvasively accelerate the rate of bone fracture healing. Although a wide range of studies has been performed, mechanism for this therapeutic effect on bone healing is currently unknown. To elucidate the mechanism of cellular response to mechanical stimuli induced by pulsed ultrasound radiation, we developed a method to apply focused acoustic radiation force (ARF) (duration, one minute) on osteoblastic MC3T3-E1 cells and observed cellular responses to ARF using a spinning disk confocal microscope. This study demonstrates that the focused ARF induced F-actin cytoskeletal rearrangement in MC3T3-E1 cells. In addition, these cells showed an increase in intracellular calcium concentration following the application of focused ARF. Furthermore, passive bending movement was noted in primary cilium that were treated with focused ARF. Cell viability was not affected. Application of pulsed ultrasound radiation generated only a minimal temperature rise of 0.1°C, and induced a streaming resulting fluid shear stress of 0.186 dyne/cm^2^, suggesting that hyperthermia and acoustic streaming might not be the main causes of the observed cell responses. In conclusion, these data provide more insight in the interactions between acoustic mechanical stress and osteoblastic cells. This experimental system could serve as basis for further exploration of the mechanosensing mechanism of osteoblasts triggered by ultrasound.

## Introduction

Bone is in a constant state of dynamic equilibrium in its mechanical loading (or reduced loading) and adopts changes in function and architecture due to these stimuli. Bone cells play a critical role in mechanosensing. Osteoblasts, known as bone-forming cells, can sense mechanical stimuli such as stress or strain [1]. A wide variety of cell-level mechanical stimuli occur due to loading, including but not limited to substrate strain, direct cellular deformation, compressive loading (increased hydrostatic pressure), intramedullary pressure and interstitial fluid flow [2], [3], [4]. These mechanical stimuli have been utilized for *in vitro* experiments to clarify the characteristics of osteoblastic responses. For example, application of fluid shear stress or mechanical strain through extracellular substrate deformation results in changes in morphology, proliferation, differentiation and gene expression that have been well investigated in osteoblasts [5], [6]. Despite the fact that many types of mechanical stimuli have been studied intensively during the past two decades, very little is known about the cellular and molecular mechanisms triggered by ultrasound in bone mechanobiology.

It is well documented that ultrasound, as a mechanical signal, can be used to accelerate the rate of bone fracture healing noninvasively in animal models [7], [8] and clinical studies [9], [10]. Histological studies suggest that ultrasound influences all major cell types involved in bone healing, including osteoblasts, osteoclasts, chondrocytes and mesenchymal stem cells. *In vitro* cell and tissue culture studies have demonstrated effects on cell differentiation and protein synthesis [11], [12]. Of note, there are three main factors that limit the study of cellular mechanisms that underlie ultrasound treatment. Above all, fracture healing is a complex physiological process, involving coordinated participation of several different cell types in addition to cell proliferation, cell differentiation, and synthesis of extracellular matrix. In this process, the combined cellular mechanisms of different cells are almost indistinguishable [13]. Secondly, the wide range of ultrasound intensities, from milliwatt to watt, have distinct effects on the bone fracture repair process through various mechanisms [14]. These effects fall into two categories, thermal effects and nonthermal effects. Nonthermal effects include acoustic cavitation, acoustic streaming and acoustic radiation force (ARF) [15]. Some of these effects may be involved in bone healing together or alone. Furthermore, the impact of ultrasound on bone depends not only on intensity, but also on frequency, pulse repetition frequency and pulse burst width as described by a number of researchers [16]–[18]. Parameters vary widely depending on the experimental design used in these reports. Thus, it is difficult to distinguish the acoustic mechanisms involved in bone healing.

The biological effects of acoustic mechanical stress (in the form of ARF) and its potential applications are commonly discussed in ultrasound study analysis. Numerous biomedical applications of ARF are related to manipulation of cells and particles in relation to standing acoustic waves. There exists a wide range of literature on ARF in standing waves used for manipulating cells in a solution, increasing the sensitivity of biosensors, separating different types of particles from a liquid or from each other, acoustical tweezers and immunochemical tests [19]–[21]. Other applications of ARF include assessment of viscoelastic properties of fluids and biological tissues [22], molecular imaging and monitoring of lesions during therapy [23]. Recently, the important roles of ARF have been proposed for ultrasound-associated promotion of fracture healing [24], [25] and enhancement in nanoparticles delivery [26], [27]. As osteoblasts are mechanosensitive, we postulated that osteoblasts may sense ARF through morphological deformation and through their surface mechanosensitive structures such as primary cilia and ion channels. Under this hypothetical assumption, forces transmitted to the cytoskeleton may influence membrane tension and curvature, thereby affecting activity of mechanosensitive ion channels, such as calcium ion channels. In addition, primary cilium projecting from the cell surface might act as a mechanosensitive structure for interaction with cytoskeleton and ion channels. Changes in intracellular calcium ion concentration function upstream of biochemical signaling cascade and trigger subsequent downstream signaling. Thus, ARF transmission to the cytoskeleton and primary cilia has the potential to stimulate activation of mechanosensitive genes and further regulate various cell functions. In order to distinguish the effects of ARF from thermal or nonthermal mechanisms, low dose and pulsed ultrasound can be used to minimize acoustic cavitation and to allow for heat dissipation between pulses [28].

In this study, we develop a methodology to allow for in-vitro mechanical manipulation of osteoblastic cells using focused ARF and then observe the morphological and calcium signaling responses. Although this ultrasound methodology differs from low intensity pulsed ultrasound (LIPUS) systems, this study represents a fundamental step towards gaining insights into the relationship between acoustic mechanical stress and the initiation of cellular responses.

## Materials and Methods

### Cell Cultures

Cells from the MC3T3-E1 mouse osteoblastic cell line (ATCC, Manassas, VA) were grown on 35 mm plastic cell culture Petri dishes in 95% air–5% CO_2_ in Dulbecco’s modified Eagle Medium (DMEM; Gibco, Grand Island, NY) which was supplemented with 20 mM HEPES and 10% heat-inactivated FBS, 2 mM glutamine, penicillin (100 U/ml), and streptomycin (100 Ag/ml) (pH 7.6). Cell medium was changed twice a week. At the time of experiment, the cells had reached approximately 85% confluency and the culture medium was changed to degassed Dulbecco’s phosphate-buffered saline (DPBS, Gibco, Grand Island, NY) which was supplemented with 1.6 mM CaCl_2_. Control samples were also subjected to the same conditions except with the omission of pulsed ultrasound exposure.

### Apparatus

**Figure 1:**
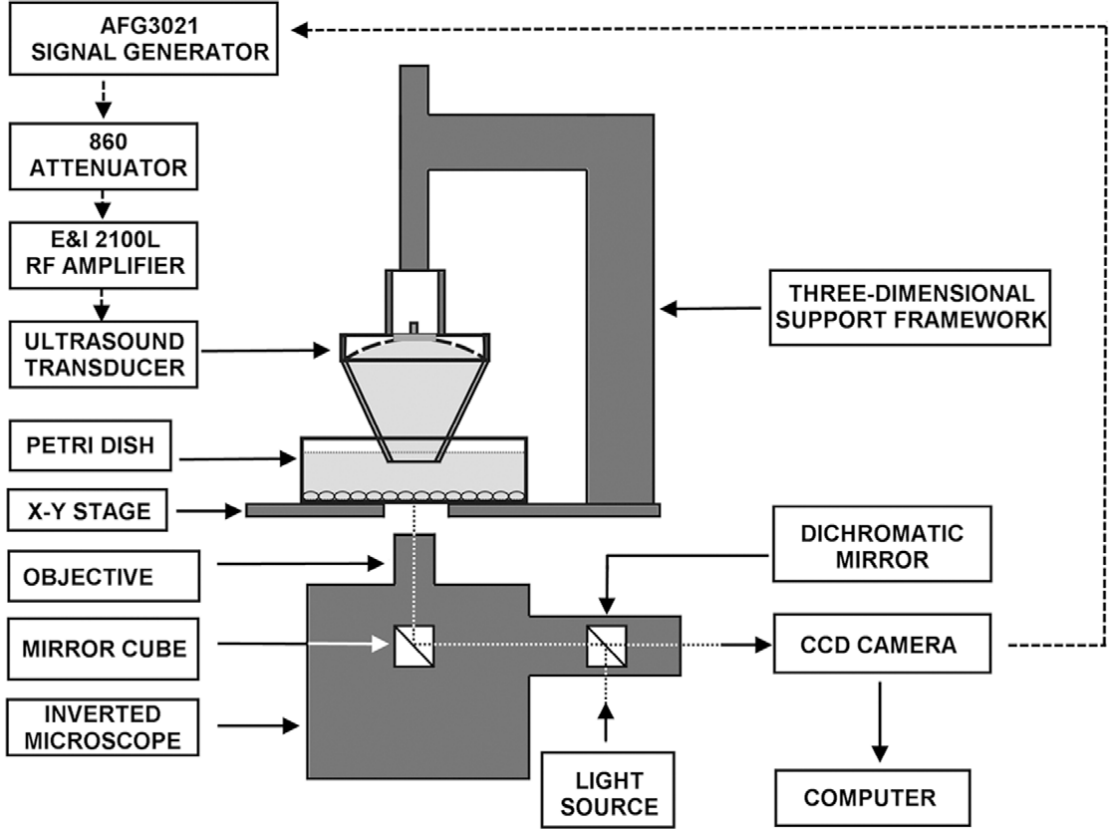
Schematic diagram of experimental setup for spatiotemporal measurements of the effects of acoustic radiation force on MC3T3-E1 cells. The setup includes an inverted microscope and a high intensity focused ultrasound system. Movement of the spherical transducer is controlled by the three-dimensional support framework.

**Figure 2:**
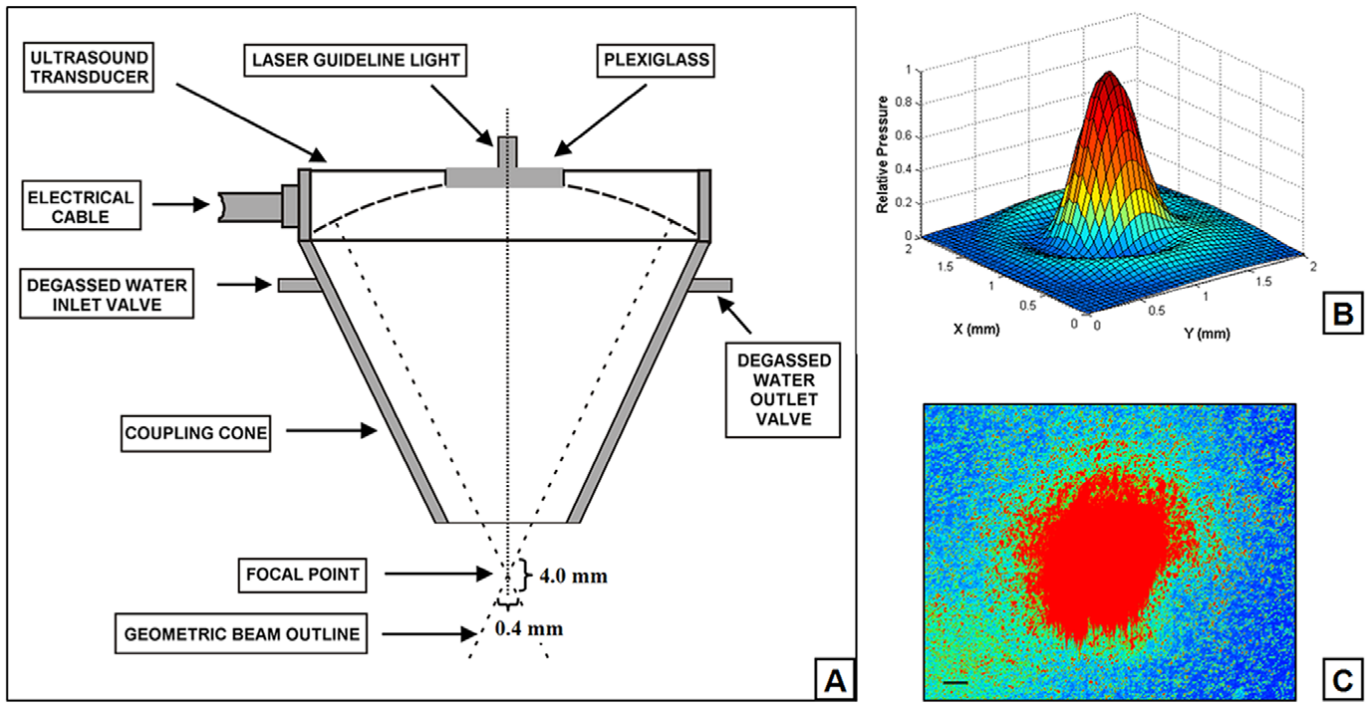
Schematic diagram of the transducer assembly. A : Spherical transducer with coupling cone and a centrally attached laser guideline light. The ellipsoidal acoustic focal zone covers approximately 4 mm axially and 0.4 mm transversely at the half-pressure points. **B**: Normalized pressure distribution through the culture dish for the single element on the radial plane at the distance of 62.75 mm of the transducer’s surface. **C**: The focal point of focused ultrasound guided by red laser light in the middle field of view of the microscope objective. Scale bar, 50 µm.

A modified high intensity focused ultrasound system (**Figure 1**) was used for the mechanobiological studies. The excitation signal generated by a waveform generator (AFG3021, Tektronix Inc, Beaverton, OR), was attenuated 10 times (using an 860 Attenuator, Kay Elemetrics Corp, Lincoln Pk, NJ) before exposure to a radio-frequency power amplifier (E&I 2100L, Electronics & Innovation, Ltd., Rochester, NY), which in turn drives a single spherical and concave transducer (H-102C, Sonic Concepts, Bothell, WA). The transducer has an active outside diameter of 64.0 mm, a radius of curvature (distance to focus from inner surface) of 62.6 mm and a central 20-mm hole that houses a piece of plexiglass and a laser module (VLM-650-03-LPA, Quarton Inc, CA), focused by a plano-convex lens with a focus of 72 mm (NT32-850, Edmund Optics, NJ). A schematic diagram of the device is shown in **Figure 1**. The focal point of the ultrasound beam was positioned at the midpoint of cells cultured in Petri dishes through a three-dimensional support framework. The transducer assembly was attached to a polycarbonate coupling cone with a 10-mm diameter exit hole. The cone was filled with degassed water and the distal end was sealed with a silicon membrane (**Figure 2A**). Prior to the experiment, the transducer was perpendicularly positioned such that the distal end of the coupling cone was submerged in the culture medium and 5 mm above the MC3T3-E1 cell monolayer, which settled on the bottom of the culture well. The beam shape at the focal zone was determined by mapping the acoustic field using a capsule-type hydrophone (HGL-0200, ONDA Corp., Sunnyvale, CA) mounted on a three-way micropositioner. At the half-power points, the focal zone was approximately 4.0 mm axially and 0.4 mm transversely (**Figure 2B**). The laser guideline light was aligned to be coaxial and confocal with the transducer. The ultrasound focal point was located in the middle field of view of the microscope objective with guidance from the laser guideline light (**Figure 2C**). The focal area was 0.126 mm^2^, so the entire field of view of the microscope (*200×200 µm at ×40, 400×400 µm at ×20*) was radiated with ultrasound. Ultrasound was turn on for one minute at the third harmonic of the transducer, 3.3 MHz with pulse duration of 300 ms, pulse repetition frequency of 0.5 Hz, duty factor of 0.15 (300 ms on and 1700 ms off). The maximum acoustic output power in this study was 6 W, the spatial averaged intensity was calculated to be 4,800 W/cm^2^ and the peak negative pressure amplitude was 9.18 MPa. Efficiency was calculated to be 68.8% [29].

To explore the thermal response to ultrasound, the radiation-induced temperature rise was recorded using a Type T (Copper Constantan) thermocouple connected to a Keithley 2000 multimeter (Keithley Instruments Inc., OH). The thermocouple was placed on the bottom of the Petri dish filled with culture medium. The study was performed using pulsed ultrasound at two power levels of 3 W and 6 W. Thermal response to continuous wave ultrasound was also measured at 6 W. All measurements were performed under the same incubation conditions as during actual ultrasound exposure.

Streaming around the cells was also investigated. Most streaming energy was blocked by the silicon membrane. Experimentally [30], local shear stress produced by the residual streaming can be calculated from local fluid velocity measurements using the following formula:
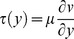
(1)where *µ* is the dynamic viscosity of the fluid, *v* is the velocity of the fluid along the boundary, and *y* is the height of the boundary. A linear shear field is a reasonable assumption as long as ∂*y* is small, preferably <10 µm. High resolution velocity measurements are therefore useful in determining the shear field at the bottom of the Petri dish. To determine the speed of local fluid, measurements of the streaming response to continuous wave ultrasound were made at three powers: 1, 3 and 6 W. The velocity of the fluid was measured by recording the movement of microspheres near the cell surface during the actual ultrasound exposure. NeutrAvidin® conjugated red fluorescent (580/605) FluoSpheres® beads (1 µm diameter; Invitrogen, Grand land, NY) were prepared and suspended in culture medium to a final concentration of 9×10^9^ microspheres/ml. Digital image recordings were captured with a high resolution digital camera (ORCA-ER-1394, Hamamatsu photonics k.k., Hamamatsu, Japan) for 30 s at 10 frames per second under the control of Slidebook™ software (version4.2, Intelligent Imaging Innovations, Inc. Denver, CO). Local fluid velocities were calculated from the measured spatial separation of imaged beads and known temporal separation.

The axial radiation force related to the acoustic power radiated by high intensity focused ultrasound was calculated for a putative total absorbing target and a holed spherical focusing transducer using the following formula,
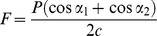
(2)where *P* is the acoustic power of a spherical zone transducer, *c* is the propagation speed of sound in the culture medium, *α_1_* is half of the internal hole aperture angle of the spherical transducer, and *α_2_* is half of the outer aperture angle of the spherical transducer [31]. In the present study, the angle of *α_1_* is 9.2° and the angle of *α_2_* is 30.7°. The speed of sound in the culture medium was about 1527 m/s at 37°C.

### Cell Viability Studies

Cell survival (live and dead cells) was determined using a live/dead viability kit (Molecular Probes, Eugene, OR) in direct culture (Control) and after 24 hours of pulsed ultrasound treatment. In brief, MC3T3-E1 cells were washed three times with DPBS. Then, the working solution containing Calcein AM and Ethidium homodimer-1 (EthD-1) was added directly to the Petri dish. Calcein AM dye (2 µM) was used to stain the cytoplasm of live cells with an intense green fluorescence and Ethidium homodimer-1 (4 µM) was used to stain DNA of dead cells with damaged membranes by producing a bright red fluorescence inside cells. After 30 min of incubation at room temperature, cells were washed once with DPBS. Next, the labeled cells were viewed under an Olympus spinning disk confocal microscope (IX-51-DSU, Olympus, Tokyo, Japan). Images were acquired to examine cell viability.

### Fluorescent Staining of Primary Cilia and Actin Cytoskeleton

To observe the morphology of primary cilia in cells, MC3T3-E1 cells were grown on coverslips and kept at confluence for at least 2 days. Cells were then incubated in 5 µM 5-chloromethylfluorescein diacetate (CMFDA, CellTracker™ Green; Molecular Probes, Eugene, OR) working solution for 30 min. The cells were then incubated in fresh DMEM for another 30 min. Next, the cells were washed 3 times with DPBS, then fixed with cold 4% paraformaldehyde for 15 min, 0.1% Triton X-100 for 10 min at room temperature and washed with DPBS 3 times. The coverslips were incubated for 30 min in 1% bovine serum albumin before incubation with primary anti-acetylated α-tubulin antibody (1∶2,000, Sigma-Aldrich, T7451) overnight at 4°C. After washing 3 times in PBS, the cells were treated with goat anti-mouse Alexa 647 secondary antibody (1∶200; Molecular Probes, Eugene, OR) in 1% bovine serum albumin for 1 h at room temperature and washed 3 times in DPBS before mounting with Vectashield mounting media containing DAPI (Vector Labs, Burlingame, CA). Cell nuclei were counterstained with DAPI. Cells were imaged on an Olympus spinning disk confocal microscope (IX-51-DSU, Olympus, Tokyo, Japan) using a 1.3 numerical aperture ×40 violet corrected objective.

To investigate the rearrangement of F-actin stress fibers resulting from pulsed ultrasound radiation, the actin cytoskeletons were stained with rhodamine-phalloidin staining. MC3T3-E1 cells were grown to subconfluence on coverslips. After one-minute of pulsed ultrasound radiation, cultures were rinsed three times with fresh DPBS for a total time of 10 min and fixed for 15 min at room temperature in PBS containing 4% paraformaldehyde, then permeabilized with 0.1% Triton X-100 for 10 min at room temperature. Cells were then rinsed three times with DPBS. After blocking with 1% BSA for 30 min, cells were incubated with Rhodamine-phalloidin (1∶100; Molecular Probes, Eugene, OR) for 20 min at room temperature. Following a brief wash, cells on coverslips were mounted on a microscope slide with mounting medium containing DAPI (Vector Labs, Burlingame, CA), and fluorescent images were acquired as described above.

### Visualization of Primary Cilia in Optical Microscope

MC3T3-E1 cells were grown on 35 mm tissue culture Petri dishes. To improve phase contrast image of cells, the transmitted light illumination column of the microscope was pushed back to a tilted position, allowing the light from the transmitted light lamp to illuminate the Petri dish, approximately 65 degrees to the horizontal axis. Cells were imaged for 60 sec (10 sec before and after 40-sec of pulsed ultrasound stimulation) at 10 frames per second under ×40 magnification using the Brightfield microscope. Images were contrast-enhanced using Image J software (NIH, Bethesda, MD).

### Fluorescence Imaging of Intracellular Calcium

To measure the changes in intracellular calcium concentration, cultured MC3T3-E1 cells were loaded with a calcium sensitive fluorescent indicator, Calcium Green™-1 AM (Ex = 506 nm, Em = 531 nm; Molecular Probes, USA), according to the manufacturer’s instructions. In short, confluent 35-mm dishes containing cells were washed twice in DPBS, and then incubated with 5 µM Calcium Green™-1 AM in DPBS at 37°C, and 5% CO_2_ concentration for 40 min protected from light. After incubation, cells were washed twice in DMEM and followed by the addition of 4 ml of fresh degassed DPBS. Cells were then prepared for observation using the Olympus spinning disk confocal microscope (IX-51-DSU, Olympus, Tokyo, Japan), so that the cells loaded well with Calcium Green™-1 could be visualized. Pulsed ultrasound radiation was performed for 1 min, and then fluorescent changes in the chosen cells were observed. Time-lapse sequences were collected on Olympus spinning disk confocal microscope every 5 s for 3 min. Fluorescence intensities were quantified using Slidebook™ software (Version4.2, Intelligent Imaging Innovations, Inc. Denver, CO).

## Results

### Temperature Changes of the Focal Region in the Culture Dish

**Figure 3:**
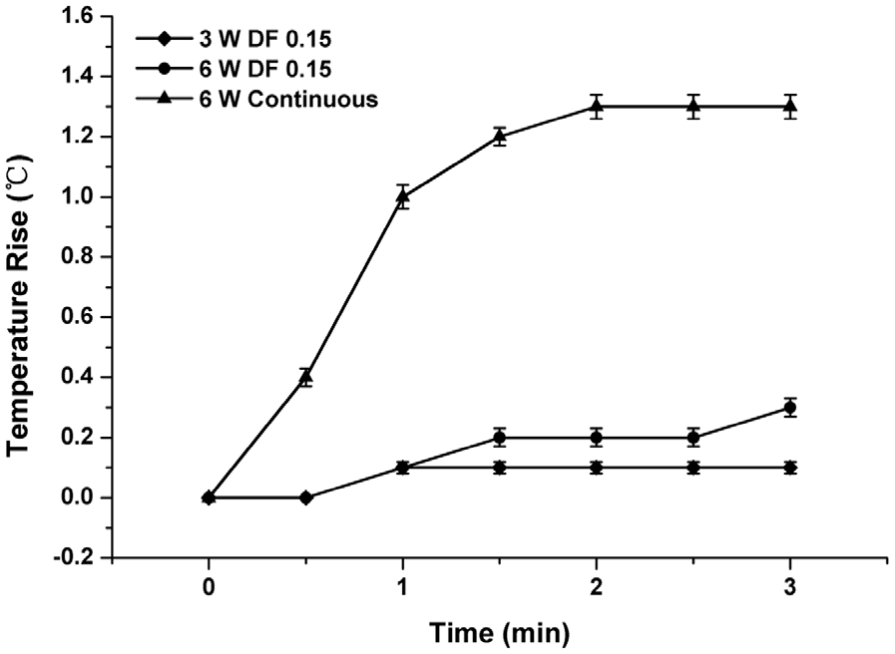
Measured average ultrasound-induced temperature rise vs. duration of different acoustical power and stimulation modes. Error bars (30-s intervals) show the standard deviation between the different measurements. Temperature was measured with a thermocouple probe immersed in the culture medium.

Ultrasound pressure distribution showed a clear peak at the center of the focal region (**Figure 2B**). Therefore, the temperature changes were specifically measured in this area of the culture dish. In the continuous radiation mode for 3 min, the results showed that the temperature of the central region of the culture dish initially rose quickly at the start of ultrasound radiation and then the increase slowed down after two minutes of radiation (**Figure 3**). The maximum rise of temperature during the radiation was approximately 1.3°C. The temperature rise was much less during pulsed radiation compared to that of continuous radiation mode, with an increase of less than 0.3°C at ultrasound power of 6 W at the end of three-minute-radiation. In addition, lower ultrasound power of 3 W caused lower temperature rise of 0.1°C. The temperature rise during ultrasound radiation may affect general metabolism and morphological structure of MC3T3-E1 osteoblasts. Hence, the pulsed ultrasound radiation mode and power to 6 W were selected for the following biological study in which the temperature rise was no more than 0.1°C over one minute radiation.

### Shear Stress and Radiation Force Induced by the Ultrasound

Fluid shear stress and radiation force caused by the ultrasound at different powers were calculated according to the formulas (1) and (2), and are listed in **Table 1**. The velocity of fluid was the average speed of 100 microspheres at various ultrasound powers. Velocity of fluid increased as ultrasound power increased. Maximum shear stress induced by ultrasound of 6 W was merely 0.186 dyne/cm^2^. Therefore, without the disturbance of nonlinear effects, such as high temperature rise and strong streaming, radiation force can be accurately evaluated by formula (2). In the present study, the maximum radiation force reached 2.497 mN and produced a pressure of 15.881 KPa with power of ultrasound at 6 W and frequency of 3.3 MHz.

**Table 1:** Calculated Dynamic Parameters for Different Acoustical Power.

Power (W)	Velocity (µm/s)	τ (dyne/cm^2^)	F (mN)	P (KPa)
1	31.737±2.133	0.022±0.001	0.416	2.646
3	140.228±13.800	0.098±0.010	1.248	7.937
6	266.046±37.306	0.186±0.026	2.497	15.881

Notes. Values for velocity and τ represent mean ± SE. The radius of the focal region is 0.2 mm. Efficiency of power conversion is 68.8%. Fluid shear stress and radiation force were calculated according to both formulas (see text for details).

### Cell Viability after Pulsed Ultrasound Treatment on MC3T3-E1 Osteoblasts

**Figure 4:**
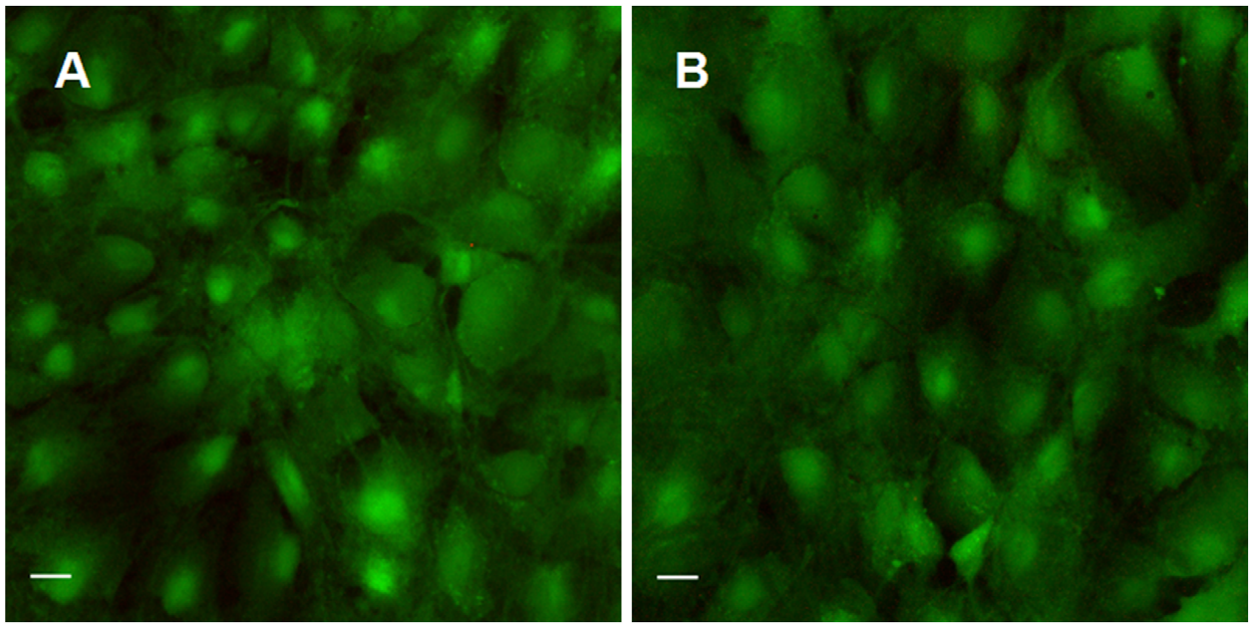
Confocal scanning images from the live/dead assay, of the MC3T3-E1 osteoblast region with pulsed ultrasound applied in Petri dishes. Compared to controls (A), viability of cell cultures assessed 24 hours post-ultrasound treatment (B) showed no significant change. Scale bar, 20 µm.

To investigate whether pulsed ultrasound radiation affects cell survival ability, the cell viability 24 hours later after ultrasound treatment was tested. Live-dead staining of MC3T3-E1 cells in Petri dish revealed that pulsed ultrasound radiation resulted in no cell death (death rate <1%) one day after radiation exposure (**Figure 4**), which is typical in a population of cultured cells. This result clearly indicates that controlled low dose ultrasound has a negligible effect on cell survival ability. Similar results were obtained by Trypan blue assay (data not shown).

### Primary Cilia Bending Movement During Pulsed Ultrasound Radiation

**Figure 5:**
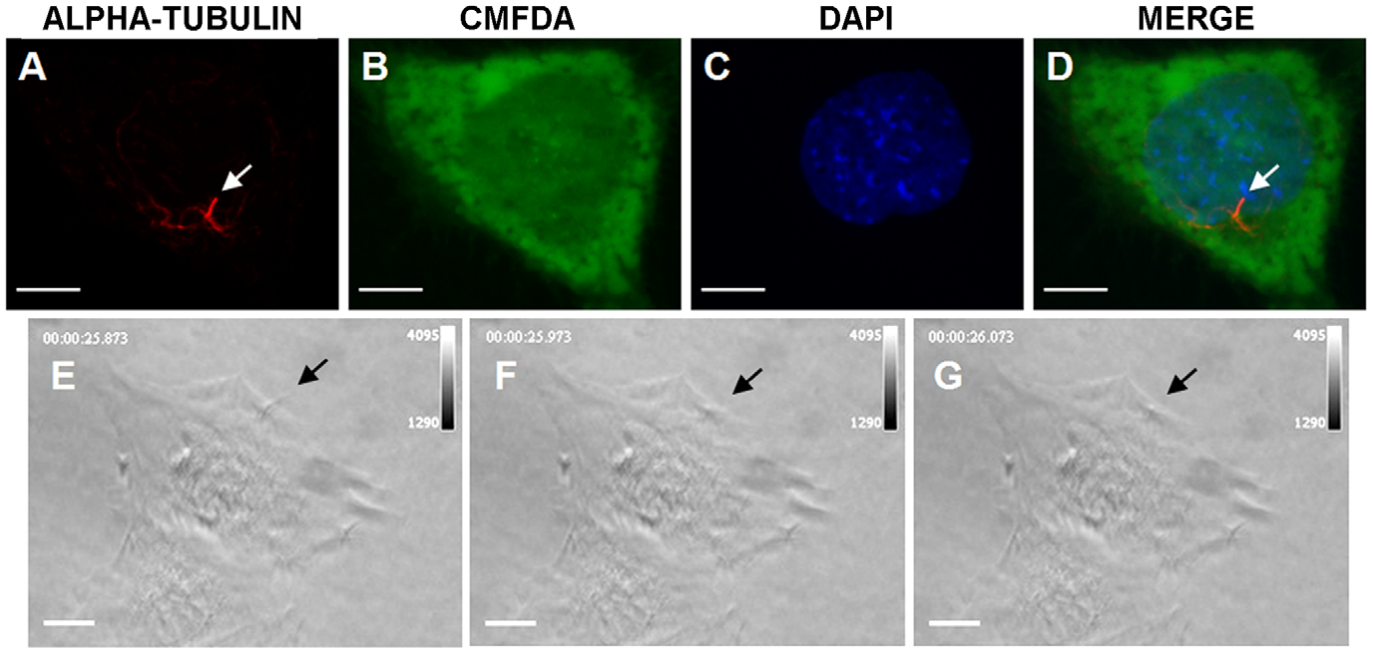
Primary cilium projections from the surface of an MC3T3-E1 osteoblast and its bending during radiation of pulsed ultrasound. The upper panel shows primary cilium stained with an antibody against acetylated α-tubulin (red) (arrowheads, A and D), cell body stained with CMFDA (green) (B and D) and nucleus stained with DAPI (blue) (C and D) in a MC3T3-E1 osteoblast. The lower panel shows the top views of bending movement of a primary cilium (arrowheads) extending from the surface of an MC3T3-E1 osteoblast before (E) and during (F and G) application of pulsed ultrasound from top to bottom. Scale bar, 10 µm.

Some studies suggested that primary cilia may play a role in mechanotransduction in bone. To determine whether primary cilia protrude from the cell surface of osteoblasts, MC3T3-E1 cells were stained for acetylated α-tubulin and CMFDA to label the cell surface, and confocal image stacks were collected (**Figure 5A to 5D**). Contrast-enhanced images revealed primary cilia extending beyond the cell surface. From a top view, primary cilia had a relatively simple linear morphology as rods (less than 10 µm long) projecting from the cell membrane of MC3T3-E1 cells (**Figure 5E**). These cilia showed corresponding movement and basal deformation when subjected to pulsed ultrasound radiation (**Figure 5E to 5G**). Passive bending movement of the cilia indicates the potential to sense ultrasound radiation force.

### F-actin Cytoskeleton Rearrangement

**Figure 6:**
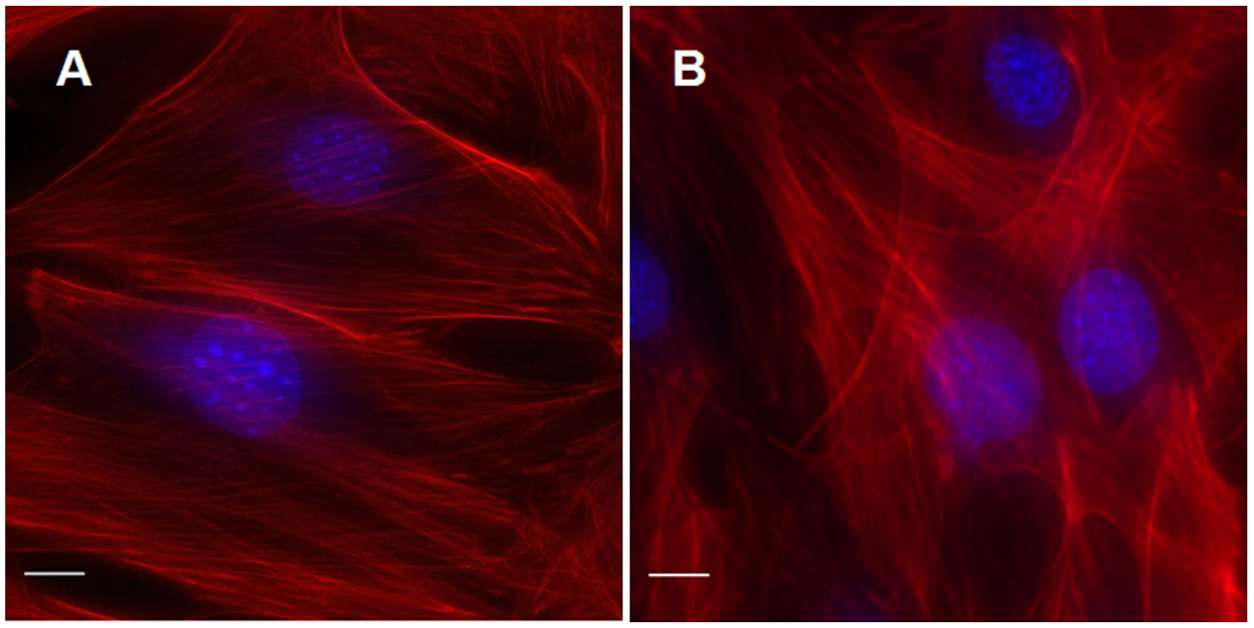
Pulsed ultrasound radiation affects the arrangement of actin cytoskeleton in MC3T3-E1 osteoblasts. Pulsed ultrasound was applied for 1 min, cells were then rinsed three times with fresh DPBS (total time,10 min), then fixed and stained with rhodamine-phalloidin. Actin stress fibers were imaged in control cells (A) and 10 min (B) after ultrasound radiation. Actin stress fibers increased following pulsed ultrasound radiation. Scale bar, 10 µm.

Rhodamine-phalloidin staining was used to monitor the reorganization of actin cytoskeletons in MC3T3-E1 cells. Representative images of MC3T3-E1 cells stained with rhodamine-phalloidin are shown in **Figure 6**. The images showed different distributions and densities of cytoplasmic actin. In control cells, the phalloidin stained images revealed typical long and straight stress fibers, composed of actin filaments during the process of cell spreading. These fibers stretch all over the cell, provide the main skeleton structure, and are responsible for the cell shape (**Figure 6A**). Changes in actin cytoskeleton were obvious after one minute of pulsed ultrasound radiation (**Figure 6B**). Pulsed ultrasound caused a substantial rearrangement of F-actin stress fibers. Enhanced fluorescence intensity F-actin stress fibers appeared in the central area of pulsed ultrasound radiated cells, compared to control cells where brighter F-actin stress fibers were only arranged along the sides of the cells. These results indicate that pulsed ultrasound affects the rearrangement of the actin cytoskeleton.

### Fluorescence Imaging of Intracellular Calcium

**Figure 7:**
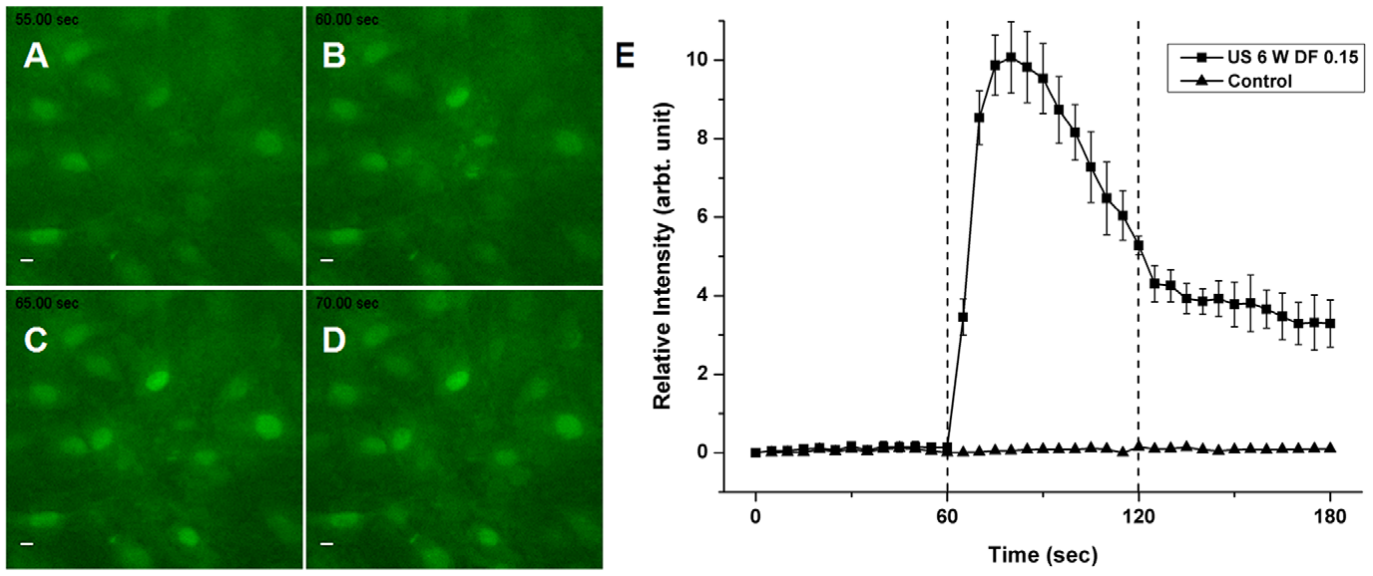
Changes in fluorescence intensities of MC3T3-E1 cells loaded with Calcium GreenTM-1. Time-lapse sequence images showing calcium influx in the cells before pulsed ultrasound radiation (A), at the start (B) of radiation and during the radiation (C and D). Compared to cells without ultrasound radiation, the pulsed ultrasound evoked a significant increase in intracellular calcium levels (E). Dotted lines represent duration of radiation on- and off-switch, respectively. Scale bar, 10 µm.

The influx of calcium ions during pulsed ultrasound radiation was monitored in real-time. Fluorescence intensities of MC3T3-E1 cells loaded with Calcium Green™-1 were increased in response to pulsed ultrasound radiation. Relative peak fluorescence intensities of cells during 1 min of ultrasound exposure were significantly increased compared to those of cells without radiation (**Figure 7**). After cessation of pulsed ultrasound radiation, fluorescence intensities gradually decreased to normal levels, indicating that pulsed ultrasound induced a transient rise in intracellular calcium concentration in MC3T3-E1 cells.

## Discussion

In this study, we developed a method to apply short-term focused ARF to the surface of individual osteoblastic cells without resultant combined effects of other acoustic phenomena. Observations of rapid cellular responses such as the passive bending movement of primary cilium, F-actin cytoskeleton rearrangement and the calcium signaling response to ARF have not been reported previously. Furthermore, cell viability was not affected.

In addition to thermal effects, ultrasound introduces several nonthermal mechanical effects, including acoustic cavitation, acoustic streaming, and ARF. Normally, cellular responses induced by ultrasound can be of both thermal and non-thermal origin [15]. Therefore, for our purposes, a fundamental step included exclusion of other phenomena resulting from ultrasound treatment of cells. In the present study, a thermal mechanism can unequivocally be ruled out. By providing ultrasound exposures using short pulses and low duty cycles, the overall rate of energy deposition for an exposure is substantially lowered so that temperature elevations are minimal (i.e., less than 0.2°C). When the duration of ultrasound radiation was shortened to one minute, this further reduced the temperature rise to no more than 0.1°C. Consequently, the cellular responses to this short-term pulse ultrasound radiation mode do involve thermal mechanism.

In addition to thermal mechanism, acoustic cavitation is another mechanism that can induce responses that result from ultrasound treatment of cells. Acoustic cavitation has been broadly described for enhancing delivery local energy to the cells, inducing cell lysis, membrane disruptions and lipid peroxidation [32]–[34]. Ultrasound energy can initiate two types of cavitation, inertial and noninertial. Inertial cavitation occurs when acoustic pressures are high enough to fracture the liquid or grow bubble nuclei. The resulting microbubbles oscillate violently a few times in response to the ultrasound before being destroyed. Noninertial cavitation refers to stable oscillations of (pre-existing) bubbles below the inertial cavitation threshold [35]. To induce cell responses, ultrasound contrast agents are typically added to enhance cavitation activity and reduce the intensity threshold at which cavitation will occur [36].

In the case of pulsed ultrasound radiation exposure used in the present study, cavitational mechanism can be precluded. Firstly, there was no ultrasound contrast agent used in this study. Instead, the working medium DPBS was degassed to remove bubble nucleation sites before radiation. This reduces the potential for cavitation in these studies. Furthermore, the extent of cavitation is influenced by the ultrasound characteristics. Higher frequency restricts the growth time of the bubbles, and pulsed ultrasound limits the number of bubbles that acquire enough energy to cause noninertial cavitation, thus allowing the bubbles to grow to their original size during the off period. Ultrasonic frequencies of greater than 1 MHz are preferred for cell manipulation, so that high pressure amplitudes can be employed without inducing ultrasonic cavitation, with its associated vigorous order-disrupting bubble activity. Crum and colleagues have shown that higher frequency of 3.5 MHz would produce much less noninertial cavitation than 1 MHz at the same pressure [37], [38]. The observation that transfection occurred at 1 MHz but not at 3.5 MHz has confirmed this opinion.

The idea that cavitation played a role in the present study is further supported by the other recent studies. Frenkel and colleagues provided evidence in two different studies that pulsed mode high intensity focused ultrasound enhanced nanoparticles delivery in murine muscle through a noncavitational mechanism [26], [27].

Acoustic radiation force is the time-average force acting on an obstacle in a sound field, which appears in the form of a steady unidirectional force even if there is no acoustic stream [39]. It is the underlying mechanism for phenomena such as acoustic streaming and acoustic fountain effects. An acoustic field propagating in a fluid medium can give rise to a bulk fluid flow termed acoustic streaming. The resulting effect of acoustic streaming is fluid shear stress. Many studies have demonstrated that osteoblasts respond to fluid flow with a change in morphology and acutely increased intracellular calcium concentration [40]–[42]. Fluid flow also promotes cell proliferation and osteogenic differentiation *in vivo* and *in vitro* [43], [44].

The effects of acoustic streaming can also be excluded in this study. We developed a sealed cone coupled with the transducer, in which most streaming energy was blocked by the silicon membrane, dramatically minimized streaming velocity on the cell surface to very lower magnitudes (on the order of hundreds of micrometers per second). Thus, the magnitude of resulting fluid shear stress was as low as 0.186 dyne/cm^2^, which is far beyond the theorized physiological range for bone cells (8 to 30 dyne/cm^2^ for physiological loading magnitudes applied at 1 Hz) [45]. Several perfusion bioreactor studies have reported that shear stresses from 0.05 to 0.3 dyne/cm^2^ were used in human stem cells culture [46]–[48]. Data showed mixed results after exposure to shear with increased calcium matrix production, upregulated expression of collagen type I and osteopontin, and accelerated osteoblastic differentiation. However, cells were exposed to long-term shear stresses from 10 to 35 days in these studies.

A hierarchical model of bone tissue structure explaining cyclic mechanical loading has shown that the sensitivity of strain detection is a function of frequency and that fluid shear stress is nearly proportional to the product of frequency and strain [45]. In the present study, there were two kinds of loading frequencies, pulse repetition frequency of 0.5 Hz and the loading frequency of ultrasound of 3.3 MHz. In regard to the former, shear stress of 0.186 dyne/cm^2^ is too small to induce any significant biological responses in one minute. However, because of the viscoelastic nature of extracellular matrix, acoustic radiation force must follow a rate dependent pattern; thus it is expected that fluid shear stress is a function of loading rate, in which the required fluid shear stress to activate cellular response would be much smaller than the low rate of loading. Therefore, regarding the latter frequency of 3.3 MHz, one-minute shear stress of 0.186 dyne/cm^2^ induced by ARF may elicit biological responses in osteoblastic cells, though further tests are required.

Although hyperthermia and cavitation are the most widely investigated ultrasound-related mechanisms that produce biological effects, the present study has established that thermal mechanism, and acoustic cavitation are not the cause of the observed cell responses. Our results indicate that pulsed radiation as used in this study induces acoustomechanical effects in osteoblastic cells through the acoustic radiation force mechanism. Studies on isolated cells have already demonstrated that ultrasound has very general effects such as plasma membrane damage [49], alterations in calcium channel activity [50], altered expression of NO synthesis [51] and up-regulation of extracellular matrix synthesis [52]. It should be noted, however, that cells can directly sense directly the effects of ultrasound-induced hyperthermia, cavitation, radiation force, streaming and resulting fluid shear stress. This experimental data may be the results of combined acoustic effects. Therefore, the novel technique developed in this study provides an opportunity to study the effects of ultrasound-induced radiation force alone. Wavelengths of ultrasound in the MHz range are much larger than the size of osteoblastic cell. As a result, in the pulsed radiation field without microbubbles, the cell undergoes only uniform compression or expansion. This type of mechanical action can be experienced by the whole cell.

A mechanosensory mechanism by which osteoblastic cells sense ARF and transmit it to biochemical signals is believed to exist, however, this mechanism is still not clearly understood. We hypothesized that osteoblasts may sense ARF through morphological deformation and their surface mechanosensitive structures. Herein, this hypothesis was preliminarily confirmed by observing the formation of stress fibers, the passive deflection of primary cilium and calcium transport within osteoblastic cells when cells were exposed to ARF.

We used a murine osteoblastic MC3T3-E1 cell culture system for the present study. These cells are capable of fully differentiating into osteoblast-like cells during culture and have been widely used in the study of bone mechanobiology. A variety of independent but interacting mechanosensory structures have been identified in osteoblasts, including the cytoskeleton and stretch-activated ion channels [53]. The cytoskeleton is a network of linked protein filaments that play key roles in the shape, motility, and mechanical properties of cells [54]. Calcium influx is implicated in numerous cellular functions such as protein secretion, differentiation, apoptosis and cell proliferation [55]. Several studies have revealed that the F-actin network and calcium ion signaling play important roles in osseous mechanobiology [41], [56], [57]. The deformed cytoskeleton can provide enhanced docking and activation sites for kinases. Disruption of the actin cytoskeleton interferes with the ability of bone cells to respond to fluid shear stress, and enhancing actin polymerization increases osteogenic differentiation [43]. Increases in intracellular calcium from both the extracellular environment and intracellular stores results from applied substrate deformation, pressure, and fluid shear [6], [58]. In the present study, our data suggested that ARF acts as a mechanical stimulus to osteoblastic cells similar to other mechanical stimuli.

There is also evidence that ultrasound can induce F-actin cytoskeleton rearrangement and calcium influx in human umbilical vein endothelial cells [59]. However, in that case, ultrasound contrast agents were used to enhance cavitation as well as the vortex-like microstreaming. The formation of stress fibers observed after ultrasound treatment may be due to the cell membrane distortion caused by ARF. One possible route for calcium release induced by ARF may be via mechanosensitive ion channels in the plasma membrane which induce release of calcium from internal stores or indirectly via the opening of hemichannels that result in ATP release, which in turn raise the intracellular calcium levels amplifying the wave propagation of calcium. This needs to be verified in future studies.

Passive deflection of primary cilium from ARF treatment is another important observation. This was confirmed in both MC3T3-E1 and MLO-Y4 cells that both possess primary cilia [60]. Although previous experiments have failed to observe primary cilia projecting extracellularly from the surface of cells using standard top view imaging techniques, our methodology provided a feasible approach for distinguishing the primary cilia by their locations and sizes using a standard top view microscope technique. Cilia were located near the nuclei and had lengths no longer than 10 µm. This result is consistent with the ciliogenesis theory and other studies using MC3T3-E1 osteoblastic cells [61], [60]. The bending movement in a passive manner of the cilia can also be detected by applying pulsed ultrasound radiation. This suggests that primary cilia may take part in sensing ultrasound in osteoblastic cells. Additionally, the basal deformation of primary cilium under ARF indicates that the existence of stretching force on the plasma membrane which may activate stretch-activated ion channels and induce F-actin cytoskeleton rearrangement. However, previous study has demonstrated that osteogenic and bone resorptive responses to fluid flow required primary cilia, and that these responses were independent of calcium influx and stretch-activated ion channels [60]. Whether primary cilia-dependent mechanotransduction resulting from ARF shares a similar mechanism to fluid flow needs to be determined in further studies.

In summary, we have developed a novel experimental system on a microscope stage for application of short-term focused ARF to osteoblastic cells. The results indicate that, in this system, the effects of pulsed ultrasound radiation are induced by ARF without significant combined effects of thermal or nonthermal mechanisms. In addition, this system does not affect cell viability. This system enables us to acquire images of cell structure deformation and calcium signaling response to the treatment with short-term focused ARF. However, it is pertinent to mention certain limitations of the present study. The study was conducted under very simple and limited conditions. The conditions of ARF, including intensity, pulse repetition frequency and pulse burst width, should be optimized for the treatment of osteoblastic cells. These experimental conditions are quite different from the clinical application systems in fracture healing, such as the LIPUS system. In addition, the key role of ARF observed in this study may not be translate directly to improved understanding of other application systems, either in vivo or in vitro. Finally, it should be noted that, in the present study, we only used a murine osteoblastic MC3T3-E1 cell culture system. Results from this single cell line may not be applicable to cells from other species. Even though the present results are but a phenomenological observation from a mechanical viewpoint, this simple experimental approach could be considered as the fundamental first step toward obtaining insights into mechanotransduction mechanism in the field of bone mechanobiology. Sophisticated mechanisms accounting for the role of cytoskeleton and primary cilia involved in this mechanobiology are currently under investigation.

## References

[1] Bikle DD (2008). Integrins, insulin like growth factors, and the skeletal response to load.. Osteoporos Int.

[2] Bloomfield SA (2001). Cellular and molecular mechanisms for the bone response to mechanical loading..

[3] Qin YX, Lin W, Rubin C (2002). The pathway of bone fluid flow as defined by in vivo intramedullary pressure and streaming potential measurements.. Ann Biomed Eng.

[4] Qin YX, Kaplan T, Saldanha A, Rubin C (2003). Fluid pressure gradients, arising from oscillations in intramedullary pressure, is correlated with the formation of bone and inhibition of intracortical porosity.. J Biomech.

[5] Jacobs CR, Yellowley CE, Davis BR, Zhou Z, Cimbala JM (1998). Differential effect of steady versus oscillating flow on bone cells.. J Biomech.

[6] You J, Yellowley CE, Donahue HJ, Zhang Y, Chen Q (2000). Substrate deformation levels associated with routine physical activity are less stimulatory to bone cells relative to loading-induced oscillatory fluid flow.. J Biomech Eng.

[7] Duarte LR (1983). The stimulation of bone growth by ultrasound.. Arch Orthop Trauma Surg.

[8] Wang SJ, Lewallen DG, Bolander ME, Chao EY, Ilstrup DM (1994). Low intensity ultrasound treatment increases strength in a rat femoral fracture model.. J Orthop Res.

[9] Heckman JD, Ryaby JP, McCabe J, Frey JJ, Kilcoyne RF (1994). Acceleration of tibial fracture-healing by non-invasive, low-intensity pulsed ultrasound.. J Bone Joint Surg Am.

[10] Cook SD, Ryaby JP, McCabe J, Frey JJ, Heckman JD (1997). Acceleration of tibia and distal radius fracture healing in patients who smoke.. Clin Orthop Relat Res.

[11] Nolte PA, van der Krans A, Patka P, Janssen IM, Ryaby JP (2001). Low-intensity pulsed ultrasound in the treatment of nonunions.. J Trauma.

[12] Korstjens CM, Nolte PA, Burger EH, Albers GH, Semeins CM (2004). Stimulation of bone cell differentiation by low-intensity ultrasound–a histomorphometric in vitro study.. J Orthop Res.

[13] Giannoudis PV, Einhorn TA, Marsh D (2007). Fracture healing: the diamond concept.. Injury.

[14] Lyon R, Liu XC, Meier J (2003). The effects of therapeutic vs. high-intensity ultrasound on the rabbit growth plate.. J Orthop Res.

[15] Dalecki D (2004). Mechanical bioeffects of ultrasound.. Annu Rev Biomed Eng.

[16] Yang KH, Parvizi J, Wang SJ, Lewallen DG, Kinnick RR (1996). Exposure to low-intensity ultrasound increases aggrecan gene expression in a rat femur fracture model.. J Orthop Res.

[17] Reher P, el-NI E, Harvey W, Meghji S, Harris M (1997). The stimulation of bone formation in vitro by therapeutic ultrasound.. Ultrasound Med Biol.

[18] Parvizi J, Wu CC, Lewallen DG, Greenleaf JF, Bolander ME (1999). Low-intensity ultrasound stimulates proteoglycan synthesis in rat chondrocytes by increasing aggrecan gene expression.. J Orthop Res.

[19] Coakley WT, Hawkes JJ, Sobanski MA, Cousins CM, Spengler J (2000). Analytical scale ultrasonic standing wave manipulation of cells and microparticles.. Ultrasonics.

[20] Lee J, Ha K, Shung KK (2005). A theoretical study of the feasibility of acoustical tweezers: ray acoustics approach.. J Acoust Soc Am.

[21] Hultstrom J, Manneberg O, Dopf K, Hertz HM, Brismar H (2007). Proliferation and viability of adherent cells manipulated by standing-wave ultrasound in a microfluidic chip.. Ultrasound Med Biol.

[22] Hollman KW, O’Donnell M, Erpelding TN (2007). Mapping elasticity in human lenses using bubble-based acoustic radiation force.. Exp Eye Res.

[23] Maleke C, Konofagou EE (2008). Harmonic motion imaging for focused ultrasound (HMIFU): a fully integrated technique for sonication and monitoring of thermal ablation in tissues.. Phys Med Biol.

[24] Rubin C, Bolander M, Ryaby JP, Hadjiargyrou M (2001). The use of low-intensity ultrasound to accelerate the healing of fractures.. J Bone Joint Surg Am.

[25] Naruse K, Miyauchi A, Itoman M, Mikuni-Takagaki Y (2003). Distinct anabolic response of osteoblast to low-intensity pulsed ultrasound.. J Bone Miner Res.

[26] Hancock HA, Smith LH, Cuesta J, Durrani AK, Angstadt M (2009). Investigations into pulsed high-intensity focused ultrasound-enhanced delivery: preliminary evidence for a novel mechanism.. Ultrasound Med Biol.

[27] O’Neill BE, Vo H, Angstadt M, Li KP, Quinn T (2009). Pulsed high intensity focused ultrasound mediated nanoparticle delivery: mechanisms and efficacy in murine muscle.. Ultrasound Med Biol.

[28] Nightingale K, Soo MS, Nightingale R, Trahey G (2002). Acoustic radiation force impulse imaging: in vivo demonstration of clinical feasibility.. Ultrasound Med Biol.

[29] Zhou Y, Zhai L, Simmons R, Zhong P (2006). Measurement of high intensity focused ultrasound fields by a fiber optic probe hydrophone.. J Acoust Soc Am.

[30] Pickard JE, Ley K (2009). Micro-PTV measurement of the fluid shear stress acting on adherent leukocytes in vivo.. Biophys J.

[31] Shou W, Huang X, Duan S, Xia R, Shi Z (2006). Acoustic power measurement of high intensity focused ultrasound in medicine based on radiation force.. Ultrasonics.

[32] Frenkel V (2008). Ultrasound mediated delivery of drugs and genes to solid tumors.. Adv Drug Deliv Rev.

[33] Barnett SB, Rott HD, ter Haar GR, Ziskin MC, Maeda K (1997). The sensitivity of biological tissue to ultrasound.. Ultrasound Med Biol.

[34] Worthington AE, Thompson J, Rauth AM, Hunt JW (1997). Mechanism of ultrasound enhanced porphyrin cytotoxicity. Part I: A search for free radical effects.. Ultrasound Med Biol.

[35] Miller DL (2007). Overview of experimental studies of biological effects of medical ultrasound caused by gas body activation and inertial cavitation.. Prog Biophys Mol Biol.

[36] Kimmel E (2006). Cavitation bioeffects.. Crit Rev Biomed Eng.

[37] Fowlkes JB, Crum LA (1988). Cavitation threshold measurements for microsecond length pulses of ultrasound.. J Acoust Soc Am.

[38] Crum LA, Roy RA, Dinno MA, Church CC, Apfel RE (1992). Acoustic cavitation produced by microsecond pulses of ultrasound: a discussion of some selected results.. J Acoust Soc Am.

[39] Vaezy S, Shi X, Martin RW, Chi E, Nelson PI (2001). Real-time visualization of high-intensity focused ultrasound treatment using ultrasound imaging.. Ultrasound Med Biol.

[40] Hung CT, Pollack SR, Reilly TM, Brighton CT (1995). Real-time calcium response of cultured bone cells to fluid flow.. Clin Orthop Relat Res.

[41] Chen NX, Ryder KD, Pavalko FM, Turner CH, Burr DB (2000). Ca^2+^ regulates fluid shear-induced cytoskeletal reorganization and gene expression in osteoblasts.. Am J Physiol Cell Physiol.

[42] Donahue SW, Jacobs CR, Donahue HJ (2001). Flow-induced calcium oscillations in rat osteoblasts are age, loading frequency, and shear stress dependent.. Am J Physiol Cell Physiol.

[43] Arnsdorf EJ, Tummala P, Kwon RY, Jacobs CR (2009). Mechanically induced osteogenic differentiation–the role of RhoA, ROCKII and cytoskeletal dynamics.. J Cell Sci.

[44] Papachroni KK, Karatzas DN, Papavassiliou KA, Basdra EK, Papavassiliou AG (2009). Mechanotransduction in osteoblast regulation and bone disease.. Trends Mol Med.

[45] Weinbaum S, Cowin SC, Zeng Y (1994). A model for the excitation of osteocytes by mechanical loading-induced bone fluid shear stresses.. J Biomech.

[46] Scaglione S, Wendt D, Miggino S, Papadimitropoulos A, Fato M (2008). Effects of fluid flow and calcium phosphate coating on human bone marrow stromal cells cultured in a defined 2D model system.. J Biomed Mater Res A.

[47] Li D, Tang T, Lu J, Dai K (2009). Effects of flow shear stress and mass transport on the construction of a large-scale tissue-engineered bone in a perfusion bioreactor.. Tissue Eng Part A.

[48] Frohlich M, Grayson WL, Marolt D, Gimble JM, Kregar-Velikonja N (2010). Bone grafts engineered from human adipose-derived stem cells in perfusion bioreactor culture.. Tissue Eng Part A.

[49] Milowska K, Gabryelak T (2005). Synergistic effect of ultrasound and phthalocyanines on nucleated erythrocytes in vitro.. Ultrasound Med Biol.

[50] Liu Y, Yang H, Takatsuki H, Sakanishi A (2006). Effect of ultrasonic exposure on Ca^2+^-ATPase activity in plasma membrane from Aloe arborescens callus cells.. Ultrason Sonochem.

[51] Hsieh YL (2005). Reduction in induced pain by ultrasound may be caused by altered expression of spinal neuronal nitric oxide synthase-producing neurons.. Arch Phys Med Rehabil.

[52] Sena K, Leven RM, Mazhar K, Sumner DR, Virdi AS (2005). Early gene response to low-intensity pulsed ultrasound in rat osteoblastic cells.. Ultrasound Med Biol.

[53] Scott A, Khan KM, Duronio V, Hart DA (2008). Mechanotransduction in human bone: in vitro cellular physiology that underpins bone changes with exercise.. Sports Med.

[54] Ingber DE, Dike L, Hansen L, Karp S, Liley H (1994). Cellular tensegrity: exploring how mechanical changes in the cytoskeleton regulate cell growth, migration, and tissue pattern during morphogenesis.. Int Rev Cytol.

[55] Berridge MJ, Lipp P, Bootman MD (2000). The versatility and universality of calcium signalling.. Nat Rev Mol Cell Biol.

[56] Malone AM, Batra NN, Shivaram G, Kwon RY, You L (2007). The role of actin cytoskeleton in oscillatory fluid flow-induced signaling in MC3T3-E1 osteoblasts.. Am J Physiol Cell Physiol.

[57] Myers KA, Rattner JB, Shrive NG, Hart DA (2007). Osteoblast-like cells and fluid flow: cytoskeleton-dependent shear sensitivity.. Biochem Biophys Res Commun.

[58] Donahue SW, Donahue HJ, Jacobs CR (2003). Osteoblastic cells have refractory periods for fluid-flow-induced intracellular calcium oscillations for short bouts of flow and display multiple low-magnitude oscillations during long-term flow.. J Biomech.

[59] Juffermans LJ, van DA, Jongenelen CA, Drukarch B, Reijerkerk A (2009). Ultrasound and microbubble-induced intra- and intercellular bioeffects in primary endothelial cells.. Ultrasound Med Biol.

[60] Malone AM, Anderson CT, Tummala P, Kwon RY, Johnston TR (2007). Primary cilia mediate mechanosensing in bone cells by a calcium-independent mechanism.. Proc Natl Acad Sci U S A.

[61] Ingber DE (2006). Cellular mechanotransduction: putting all the pieces together again.. FASEB J.

